# Spontaneous prematurity in fetuses with congenital diaphragmatic hernia: a retrospective cohort study about prenatal predictive factors

**DOI:** 10.1186/s12884-017-1652-6

**Published:** 2018-01-12

**Authors:** Bruna Maria Lopes Barbosa, Agatha S. Rodrigues, Mario Henrique Burlacchini Carvalho, Roberto Eduardo Bittar, Rossana Pulcineli Vieira Francisco, Lisandra Stein Bernardes

**Affiliations:** 1 Departamento de Obstetrícia e Ginecologia, Faculdade de Medicina, FMUSP,, Universidade de São Paulo, São Paulo, Av. Dr Eneas de Carvalho Aguiar, 255 Cerqueira Cesar, São Paulo, CEP: 05403-000 Brazil; 2 Estatística, Departamento de Ginecologia e Obstetrícia, Faculdade de Medicina, FMUSP, Universidade de São Paulo, Av. Dr Eneas de Carvalho Aguiar, 255 Cerqueira Cesar, São Paulo, CEP: 05403-000 Brazil

**Keywords:** Predictive factors, Prematurity, Congenital diaphragmatic hernia, LHR

## Abstract

**Background:**

To evaluate possible predictive factors of spontaneous prematurity in fetuses with congenital diaphragmatic hernia (CDH).

**Methods:**

A retrospective cohort study was performed. Inclusion criteria were presence of CDH; absence of fetoscopy; absence of karyotype abnormality; maximum of one major malformation associated with diaphragmatic hernia; ultrasound monitoring at the Obstetrics Clinic of Clinicas Hospital at the University of São Paulo School of Medicine, from January 2001 to October 2014. The data were obtained through the electronic records and ultrasound system of our fetal medicine service. The following variables were analyzed: maternal age, primiparity, associated maternal diseases, smoking, previous spontaneous preterm birth, fetal malformation associated with hernia, polyhydramnios, fetal growth restriction, presence of intrathoracic liver, invasive procedures performed, side of hernia and observed-to- expected lung to head ratio (o/e LHR). On individual analysis, variables were assessed using the Chi-square test and the Mann-Whitney test. A multiple logistic regression model was applied to select variables independently influencing the prediction of preterm delivery. A ROC curve was constructed with the significant variable, identifying the values with best sensitivity and specificity to be suggested for use in clinical practice.

**Results:**

Eighty fetuses were evaluated, of which, 21 (26.25%) were premature. O/e LHR was the only factor associated with prematurity (*p* = 0.020). The ROC curve showed 93% sensitivity with 48.4% specificity for the cutoff of 40%.

**Conclusion:**

O/e LHR was the only predictor of prematurity in this sample.

## Background

Congenital diaphragmatic hernia (CDH) is a severe fetal malformation, with an incidence of 1–2200 live births [1]. CDH is characterized by non-development of the diaphragmatic membrane which can lead to devastating post-natal consequences, particularly pulmonary hypoplasia and pulmonary hypertension, having a mortality of around 50–60% [2]. Premature birth is a significant risk factor for neonatal morbidity and mortality in most diseases, being directly or indirectly responsible for 75–88% of all perinatal mortality in newborns [3].

Studies evaluating the incidence of prematurity in fetuses with CDH have shown rates varying from 22.4 to 36% [4–7]. These studies also revealed that survival of premature newborns with CDH was lower than that of term infants (OR 3.06–3.20 for mortality in premature newborns) [5, 6]. Thus, prematurity is an important prognostic factor for fetuses with CDH.

Considering the importance of prematurity for this population, it would be valuable to identify possible prenatal predictive factors and in the future to study interventions that could lower this rate. Thus, the aim of the present study was to evaluate possible predictive factors for spontaneous prematurity in fetuses with CDH.

## Methods

A retrospective cohort study including 80 fetuses with congenital diaphragmatic hernia was performed at the Fetal Medicine Center of the Obstetrics Department of ‘Hospital das Clinicas’ of the University of São Paulo School of Medicine in São Paulo, Brazil, from January 2001 to October 2014. Hospital databases, which are systematically filled after each evaluation and have not been changed during the period of the study, were used in order to obtain information regarding follow up.

Inclusion criteria were: single pregnancy, live fetus at birth; presence of CDH; maximum of one major malformation associated with hernia (only malformations that could be postnatally treated or minor defects without clinical significance were included); absence of karyotype abnormality (either prenatal karyotype or post-natal evaluation by a pediatrician); absence of fetoscopy (FETO); absence of medical indication for premature delivery and complete follow-up data available in the database or informed by telephone.

The study was approved by the Institutional Review Board of the University of São Paulo (CAPPesq, 325.907).

Gestational age was calculated based on last menstrual period (LMP) in accordance with the first trimester ultrasound or, if unavailable, with the early second trimester ultrasound. Preterm delivery was defined as a delivery prior to 37 weeks’ gestation.

Variables analyzed were: maternal age (in years); primiparity (y/n); associated maternal disease, considered as the presence of any disease during pregnancy (y/n); smoking during pregnancy (y/n); previous spontaneous preterm birth (y/n); presence of any malformation associated with hernia (y/n); polyhydramnios defined as Amniotic Liquid Index (ILA) > 25 cm or deepest pocket ≥8 cm (y/n); fetal growth restriction, defined as estimated fetal weight less than the 10th percentile of Hadlock [8] (y/n); invasive procedures performed (including chorionic villus sampling, amniocentesis, cordocentesis, amniotic fluid drainage and thoraco-amniotic shunt)(y/n); side of hernia (left/ right); observed to expected lung to head ratio measured by the longest diameter method (o/e LHR). The observed LHR was measured by the method of the largest diameter and the o/e LHR was calculated as described by Jani et al. [9], 2012.

Statistical analysis was performed using the SPSS 20.0 for Windows program. Categorical variables were described using relative frequencies (percentages) and absolute frequencies (n) while statistical analysis was performed using means of Chi-square analysis and Fisher’s exact test where appropriate. Numeric variables were submitted to the Kolmogorov-Smirnov test to evaluate data distribution, and since data was non-parametric, the Mann-Whitney test was used to compare values in premature and term groups. Median, minimum and maximum values were expressed to indicate the variability of the data. After individual analysis, a multiple logistic regression model with stepwise variable selection method was applied to select variables that could individually predict preterm delivery. Since we aim to evaluate the relationship between the different variables studied in the stepwise analysis, a significance level of 10 % was established for inclusion of the variable in the model and 0.2 for its removal. The Hosmer and Lemeshow test was performed to evaluate the goodness of fit of the model. A ROC curve was constructed with the significant variable, identifying values with best sensitivity and specificity to be suggested for use in clinical practice. The results were considered statistically significant for values of *p* < 0.05.

## Results

**Fig. 1:**
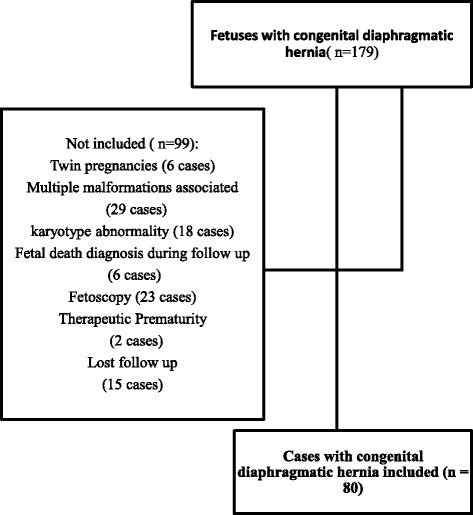
Flow Chart of the study population

Figure 1 characterizes the study population.

Eighty patients met the inclusion criteria, of which 59 (73.7%) had a term delivery and 21 (26.2%) a spontaneous preterm delivery.

Median gestational age at first follow-up at the service (first ultrasound) was 29.4 weeks of gestation (range 18.3–39.3 weeks). Median gestational age at delivery was 37.8 ± 5.5 weeks (range 29.6–40.8 weeks).

The median gestational age at the o/e LHR was 31.2 weeks (range 18.7–37.8 weeks). Of the 80 patients included in the study, 56 (70%) had at least one measure of LHR during pregnancy.

Table 1 describes the parameters evaluated individually and their percentage in preterm and term births.

**Table 1:** Relationship between different variables and prematurity in fetuses with Congenital Diaphragmatic Hernia – Univariate Analysis - January 2001-October 2014

	Preterm birth (*n* = 21)	Term birth (*n* = 59)	*p*
Maternal age, median (min-max)	27 (18–42)	24 (16–41)	0,056
Primiparity, n (%)	7 (33,3)	28 (47,45)	0,203
Associated maternal diseases, n (%)	3 (14,3)	11 (18,65)	0,751
Smoking during pregnancy, n (%)	0 (0)	2 (3,38)	0,999
Previous spontaneous preterm birth, n (%)	2 (9,5)	1 (1,7)	0,167
Malformations associated with, n (%)	8 (38)	19 (32,2)	0,789
Polyhydramnios, n (%)	7 (33,3)	25 (42,4)	0,606
Fetal growth restriction, n (%)	5 (23,8)	18 (30,5)	0,780
Invasive procedures performed, n (%)	11 (52,4)	21 (35,6)	0,203
Side of hernia, n (%)	17 (81)	48 (81,35)	0,999
o/e LHR, median (min-max)*	30,11 (17,2–42,5)	37,55 (11,3–93,1)	0,045

* *n* = 15 (preterm births), *n* = 41 (term births)

After the initial analysis, a multiple logistic regression model was adjusted considering the outcome prematurity as the dependent variable and the following variables as covariates: maternal age, primiparity, associated maternal diseases, previous spontaneous preterm birth, malformations associated with hernia, polyhydramnios, fetal growth restriction, presence of intrathoracic liver herniation, invasive procedures performed, side of hernia and o/e LHR. After selecting variables, only o/e LHR was associated with prematurity (*p* = 0,02) and was the only covariate that remained in the final model. The o/e LHR was considered an independent predictor of preterm delivery in fetuses with CDH. The Hosmer and Lemeshow test *p* value was 0.345, indicating good fit of the model. Table 2 shows the results of the final model.

**Table 2:** Results of final model estimating probability for prematurity in fetuses with Congenital Diaphragmatic Hernia - January 2001 to October 2014

Variable	Coefficient ($\widehat{b}$)	Odds Ratio (OR)	OR (CI 95%)	*p*
Intercept	1.188	3.282	(1.369–7.861)	0.174
o/e LHR	−0.059	0.943	(0.919–0.966)	0.020

The estimated probability of premature birth, $\widehat{p}$, is given by:

$$ \widehat{p}=\frac{\exp \left({\widehat{b}}_0+{{\widehat{b}}_1}^{\ast } LHRo/e\right)}{1+\exp \left({\widehat{b}}_0+{{\widehat{b}}_1}^{\ast } LHRo/e\right)}, $$

This formula shows that for each one unit decrease in o/e LHR there is a 6.38% increase in the risk of preterm birth.

**Fig. 2:**
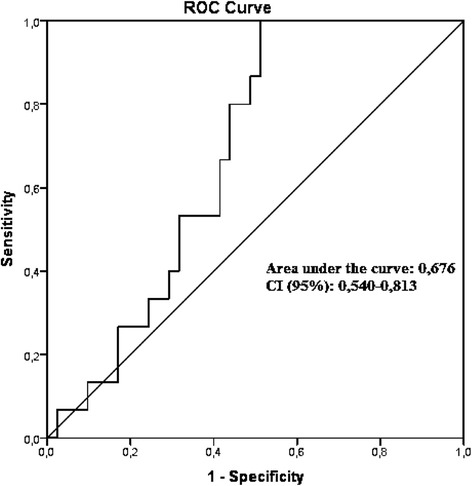
ROC Curve for O/E LHR in the prediction of premature birth in fetuses with diaphragmatic hernia

The ROC curve (Fig. 2) shows the sensitivity and specificity for different cut-offs of o/e LHR and for prediction of preterm delivery in fetuses with CDH. The area under the curve is 0.676 (IC 0.540–0.813) and the p value was 0.045, rejecting the null hypothesis of the AUC is 0.5. Table 3 shows the sensitivity and specificity for some cut-off points of o/e LHR.

**Table 3:** Sensitivity and specificity of o/e LHR and prediction of prematurity in fetuses with Congenital Diaphragmatic Hernia - January 2001 to October 2014

o/e LHR (%)	Sensitivity (%)	Specificity (%)
20	6.7	95.1
25	26.7	78.0
30	46.7	68.3
35	73.3	56.1
40	93.3	48.8
45	100	43.9
50	100	39
55	100	31.7

## Discussion

After multiple regression analysis, the observed/expected LHR was the only predictive factor of preterm delivery in fetuses with CDH in our population. O/e LHR is used in clinical practice to prenatally evaluate post-natal prognosis where lower o/e LHR is associated with worse post-natal prognosis, predicting higher mortality rates [10, 11] and worse pulmonary hypertension [10].

It is unknown what pathophysiological mechanism is associated to the higher preterm birth rates observed in fetuses with lower o/e LHR. Some studies report that CDH is associated with higher frequency of polyhydramnios [12, 13]. One of our first hypotheses was that this association would cause greater uterine distension and predisposition to prematurity. However, this association remains controversial and in our population preterm delivery was not higher in fetuses with polyhydramnios than in fetuses with normal amount of amniotic fluid, making this hypothesis less likely.

Another possible explanation is that there is an association of prematurity with the pulmonary hypoplasia present in fetuses with lower o/e LHR. Purisch et al. [6] evaluated the risk of preterm delivery in fetuses with eight different isolated malformation and fetuses with more than one associated malformations and found that renal agenesis was the malformation that had the highest risk for preterm birth (OR 6.1, 4.3–8.5, 95% CI). Renal agenesis is not associated with uterine distension. In fact, the condition generally leads to smaller uterine height in pregnancies without fetal malformation due to the presence of oligoamnios, as well as to a higher degree of pulmonary hypoplasia [14]. There may be signaling factors in fetuses with pulmonary hypoplasia that predispose them to premature birth.

In 2013 Ali et al. [15], published prematurity outcomes in fetuses submitted to FETO. Their study showed that fetuses that delivered <35 weeks had lower observed/expected LHR than those that delivered >35 weeks (0.16 vs 0.21, *p* = 0.01). Although the authors interpreted it as a risk factor for post-natal death (as it is), it may also signify an increased a priori risk for prematurity, and this risk might be considered for parental counseling and interventions that could interfere with prematurity rates.

The incidence of overall prematurity in our study was 26.2%, which is similar to rates in previously published studies (36%, 30 and 22.4% in the studies of McGivern et al.(7), Levison et al. [5] and Tsao et al. [4] respectively). Similarly Purisch et al. [6], evaluating prematurity rates in fetuses with different malformations, observed that 23.1% of fetuses with CDH were premature.

Unlike Purish et al. [6], we did not observe a higher incidence of prematurity in the presence of other malformations associated with hernia. In the study of Purish, the incidence of prematurity in fetuses with isolated CDH was compared to the incidence of prematurity in fetuses with multiple malformations. The relative risk of preterm delivery in fetuses with multiple malformations was higher (adjusted OR 7.2, 4.7–11 95% CI) than in fetuses with isolated CDH (adjusted OR 3.2, 2.4–4.3, 95% CI). The cited study, however, considered all fetuses with multiple malformations, including those without CDH, and this may account for the differences when compared with our results.

A limitation of the present study was that, being a retrospective study, other factors associated with prematurity described in the general population, such as race, socioeconomic status, maternal psychological characteristics, family history, cervix length, genitourinary infection, vaginal bleeding or frequent contractions during the pregnancy and changes in the cervix could not be analyzed. Also, the median gestational age at first evaluation was 29.4 weeks (probably reflecting the late diagnosis and referral in our country), and, as a retrospective study, the influence of care provided by different team members can’t be assessed. We believe that these are very preliminary results and all these potential factors should be tested with a prospective and multicenter study comparing results against those observed in our study.

A benefit of individual risk assessment of prematurity in fetuses with CDH is the possibility of promoting preventive measures for those fetuses with increased risk. In this case, it would be advantageous to use cut-offs with high sensitivity, albeit with intermediate specificity. The o/e LHR of 45% would yield high sensitivity, and further studies could test preventive treatments, such as progesterone use, in this high-risk population. The use of progesterone is widely reported for other risk factors, such as history of prematurity [16], but it has not been studied in fetuses with malformations.

Further studies are needed to consolidate the outcome of this study, includind also other markers of pulmonary hypoplasia such as fetal lung volume estimated by magnetic ressonace, and also to test therapeutic measures that may be used in clinical practice to prevent prematurity in these fetuses.

## Conclusions

Our study is the first to show o/e LHR as a predictor of spontaneous preterm delivery in fetuses with CDH. If confirmed, this finding paves the way for individual risk assessment of women in this subgroup of patients, allowing the opportunity for preventive measures to be taken.

## Abbreviations

CDH: Congenital diaphragmatic hernia
FETO: Fetoscopy
ILA: Amniotic Liquid Index
LMP: Last menstrual period
O/eLHR: Observed-to- expected lung to head ratio
